# Making progression and award decisions

**DOI:** 10.15694/mep.2017.000200

**Published:** 2017-11-07

**Authors:** Steven Ashley Burr, Vehid Max Salih, Thomas Gale, David Moles, Terry Vallance, David Bristow

**Affiliations:** 1 Peninsula Schools of Medicine & Dentistry

**Keywords:** assessment, educational governance

## Abstract

This article was migrated. The article was marked as recommended.

It is incumbent on medical schools to show, both to regulatory bodies and to the public at large, that their graduating students are “fit for purpose” as doctors. Since students graduate by virtue of passing assessments, it is vital that schools quality assure their assessment procedures, standards and outcomes. An important part of this quality assurance process is how progression and award decisions are made. This begins with developing clear evidence-based policies and processes that ensure assessments are effective, relevant, fair, robust and secure. Assessment is a series of processes primarily designed to enable judgements to be made as to whether a student has, or has not, met the standard required for the outcomes at that stage. This article will provide a clear rationale and guidance for establishing robust processes for making progression and award decisions.

## Introduction – Purpose

“Intermediate and high-stakes decisions are based on multiple data points after a meaningful aggregation of information and supported by rigorous organisational procedures to ensure their dependability.” ( Van Der Vleuten et al., 2015). Our article describes those “rigorous organisational procedures” which are necessary when making decisions. The governance and quality assurance of assessment delivery, results processing, and decision making on student outcomes is an area of critical importance to ensure that only those students who meet the necessary requirements are permitted to proceed to practice. Thus, not only do we wish to identify and stop those candidates who have not met the requirements, but we also want to identify and progress those candidates who have met the requirements. In the UK, the General Medical Council has previously highlighted considerable variability and some confusion amongst medical schools surrounding progression policies ( GMC, 2014), which is consistent with findings more widely across the sector ( Stowell, 2004; Yorke, et al., 2008), and has led to students being incorrectly graduated ( BBC, 2009). The UK Code for Higher Education stipulates that for groups making progression and award decisions: membership, procedures, powers and accountability all need to be clear ( QAA, 2013b; Indicator 15); the regulations must be applied fairly ( QAA, 2013b; Indicator 16); and, decisions recorded accurately and communicated promptly to students ( QAA, 2013b; Indicator 17). Particular emphasis is placed by the QAA on: lines of accountability; regulations which are applicable; inclusion of both internal and external examiners; recording the views of those who are absent; identification and management of meetings that are not quorate; limits of chair’s action; consistent application of discretion (e.g. around extenuating circumstances and borderline cases); and, identification and management of conflicts of interest ( QAA, 2013b). Aspects of progression that have been indicated to require more clarity by the GMC include the: maximum time permitted to complete a programme; number of reassessments permitted; separate handling of extenuating circumstances; and, support for ‘exit with grace’ arrangements ( GMC, 2014). We aim to provide guidance on how to manage the procedures that confirm assessments: are fair; measure what they claim to a consistent standard; and, are appropriate for the award of credit and qualification.

## Separate the confirmation of assessment results from student outcomes

It is important to separate decisions on the process affecting a cohort from decisions on individual students. Across the sector this is often achieved by creating a physically and temporally separate two tier examination board system ( QAA, 2015). Decisions about the validity of individual summative assessments are made by committee, with oversight and input from an external examiner. Decisions about individual students’ awards are made by a second committee, with oversight and input from another external examiner. For practical reasons, it might be appropriate for the two meetings to be joined into one. The first half of the meeting reviews the standard of the assessment process and confirms the results for all students taking all assessments in the module or stage. Based on those results, the second half of the meeting decides the overall outcome and award for each student. Decisions about an assessment or student outcome, which have been made in this way, cannot be changed later by any one individual member of staff, only by the committee concerned, and would require a formal appeal to trigger reconsideration. To change the outcome, the appeal would need to contain evidence that there had been: (1) a processing error or some other procedural irregularity (e.g. transcription error) that could have materially affected the decision; (2) a manifestly unreasonable decision (e.g. discounting a retrospective claim for an otherwise valid extenuating circumstance when it could not reasonably have been disclosed earlier); or, (3) a biased or prejudiced (i.e. demonstrably unfair) decision (e.g. an examiner not marking impartially).

## Have a comprehensive agenda

The agenda and supporting documentation should be checked by the chair and secretary in advance of any meeting. On the day, it is necessary to first confirm: the meeting is quorate, the identity of voting members, that all those present declare any potential conflicts of interest, and that all details discussed at the meeting are treated confidentially. Measures should be taken so that anonymity of students is maintained throughout all documentation presented to avoid potential bias amongst panel members. It is necessary to review any action points from the last meeting, along with the last external examiner’s report, the school response, and any further matters which the external examiner wishes to raise. It is then useful to summarise the regulations in place for the award of credits and progression decisions.

To decide on the validity of an assessment there should be review of any invigilator and academic offence reports, and statement (by the academic lead for the assessment) of the processes that have been undertaken to confirm the accuracy of the data. This should be followed by a psychometric review ( Coombes et al., 2016) of descriptive statistics, standard setting, reliability, and demographics (to safeguard equality); with activities based on threshold for action policies in each case. There should be confirmation of any questions removed from the assessments post-hoc with justification and, where appropriate, review of possible reasons for any changes in any aspect of performance (e.g. pass rate) between cohorts.

Points to consider when making decisions on outcomes for individual students include:

• Equality (the adequacy of any alternative provision and additional support, e.g. for disability);
• Health and fitness to study (e.g. immune status, occupational health review) considering any potential impact on the quality of the programme and welfare of other students;
• Valid extenuating circumstances (i.e. short-term, unexpected, outside control of student, and with potential to adversely affect performance), which must have been validated separately prior to the meeting;
• Fitness to Practice and concerns about conduct (which should include students who have withdrawn in order to satisfy duty to report negative outcomes to the national excluded student database; MSC, 2015).

The academic lead for each assessment then has the opportunity to report issues (i.e. changes since last meeting and any interim problems). This is followed by the review of results. Efficiency can be increased by considering students with no issues collectively; separate from the individual consideration of students with specific issues (e.g. valid extenuating circumstances, incomplete remediation, or repeat status). The outcome must be agreed and recorded for each student and where applicable, deferrals and referrals clearly identified.

Following the decisions at each meeting, the external examiner is required to comment and formally confirm the outcomes. It is then prudent to confirm their remaining tenure. The date of the next meeting should be confirmed and, if appropriate, the results release date. When releasing results it is necessary to ensure faculty support is in place for students in difficulty, so results are not normally released near the end of the working day, at the end of the working week, or during a holiday. If a student’s studies are being terminated then the breaking of such news should ideally be in a face to face personal meeting if feasible (with explanation supported by a handed letter that includes signposting to additional support, careers counselling and rights of appeal), and a separate communication to any formal sponsors as appropriate. It is necessary to close the business of the meeting formally, acknowledging any prior agreement to postpone any specific decisions conditional on the availability of additional information.

## Safeguard student anonymity

All student records should be anonymised to minimise unconscious bias and ensure decisions remain impartial of student personal details (including student id numbers). Similarly, there should be no representations from individual students or their advocates, as differential lobbying could bias outcomes. To obviate transposition errors during anonymization it is advisable to use standard software functions to hide/reveal columns of both names and student numbers, and to re-order students by their performance rank. Students should then be listed with the same row number in the spreadsheets for any subsequent meeting or consideration. Where there are students whose cases are not a straightforward decision to progress or receive an award, then it is necessary to minute the holistic consideration of each anonymous individual in turn.

## Be clear on the decision making process

The key members forming the quorum are the relevant senior academic, senior administrator, and external examiner. The senior academic’s role is to chair the meeting and ensure the adequacy of the data presented on which decisions will be based. The senior administrator’s role is to ensure that the regulations are adhered to and that agreed decisions are unambiguously recorded. The external examiner’s role is to ensure minimum threshold standards are maintained for the award that are comparable to elsewhere, and that students are treated fairly. In cases of unavoidable absence, each may take part remotely provided there is simultaneous communication and equivalent access to information. Alternatively, a delegated nominee approved by the programme lead, or academic of higher authority may substitute key staff members. Other staff with expertise relevant to the decisions being made should attend and can be voting members. The meetings can also be observed by other academic or quality assurance staff to develop their understanding of the process. Members eligible to vote should be identified, and restricted to academic staff because the decisions are of academic judgement. The external examiner should not vote, and the chair should retain a casting vote or vote last, lest they bias the judgements made by others.

The decision about each student requires a review of their individual performance across assessments and confirmation of their outcome according to the regulations. Typically, the decision options are to convey credit and either recommend an award or permit progression, or to place requirements on the student. Requirements may include, but not be restricted to: (1) repeat the year (confirming whether as a first or second attempt, and that they have not exceeded any limit for repeats); (2) undertake additional assessment (i.e. resit/referred) within the same year (with date and external examiner involvement prior to reconvening a meeting to make a decision); (3) suspend (if disciplinary); (4) interrupt (if not disciplinary); or, (5) withdraw (while also confirming the award of any credits or alternative exit qualification). When the regulations do not provide a precedent then decisions should be reached by majority vote. Each case should be judged on its individual merits, bearing in mind natural justice and processes that a reasonable person would understand and relate to as fair. In a borderline case, it can be useful to consider whether there is any evidence that the performance of the student is improving or not.

All assessment results conveyed to students during the year are provisional until ratified at the appropriate meeting, and once ratified, decisions cannot be overturned by any individual. Thus, ‘chair’s action’ cannot be made unilaterally, as all decisions are group decisions. As such, any action to be taken by a chair needs to be explicitly pre-agreed by the group. For example, ‘if X is achieved then Y will be conferred’, and then ‘if X is not achieved then a decision will have to be referred back to the group’. Therefore, it is necessary to distinguish clearly between having agreed a decision and ‘referring’ a student for a recommendation, or ‘deferring’ a decision until later if further information is required.

## Remember the need to be fair to all

To confirm the fairness of an individual assessment it is necessary to ask whether there is sufficient evidence that the assessment is: (1) appropriate in method and content, (2) adequately explained, (3) applied equitably, and (4) capable of remediation.

(1) *To be appropriate in both method and content*, the ascribed learning outcomes must be relevant to the programme, testable, and what the student is asked to do must be achievable. Even if all students pass then it should be confirmed that the assessment was capable of discriminating those who should fail. (2) *To be adequately explained* to students in terms of both purpose and process then the philosophical rationale and a full formative attempt, should both be made available prior to requiring the first summative attempt at that assessment type. Summative deadlines should be clearly publicised in advance, without the expectation of reminders, and strictly adhered to. A student who leaves tasks until near the end of the time allowed is implicitly accepting the risk that things outside their control may prevent them completing what is required. (3) *To be applied equitably* to all students it is important that decisions are not influenced by outcomes, such as progression nor retention rates, in order to be fair to students who happen to be in strong or weak cohorts ( Stowell et al., 2015). If there is differential attainment between protected demographic groups then there should be consideration as to whether a threshold for action has been reached, and then whether a cause has been identified which would justify moderation ( Burr & Leung, 2015). Similarly, be vigilant for circumstances that lead to double jeopardy, whereby one adverse judgement leads to another supposedly independent adverse judgement. When determining standards (e.g. pass, merit, or distinction) look for opportunities to correlate final performance with external indicators such as national examinations in order to supplement the evidence base supporting the methodology for determining awards. (4) *It is necessary to ensure that there is equal opportunity to remediate* between failed assessments. For example, there must be adequate time for remediation between releasing results and the next assessment.

If a point of variation in assessment conditions is reported, then it is necessary to decide whether the standard has been compromised and students have been advantaged or disadvantaged. For example, if a student was given a prompt by an examiner that other students did not receive, then that student may have been advantaged. Nevertheless, if the correct practice would have been not to give the prompt, then re-running the exam for those students who were comparatively disadvantaged could be of no benefit to them, as the assessment would have to be re-run correctly without the prompt again in order to maintain the standard being tested. Thus, the students would receive the assessment under the same conditions as that they had already taken. However, if the prompt compromised the standard being tested then the student who was potentially advantaged should be required to take the assessment again. Consider another example: if a student had not received information given to other students that could have made a question easier, and the student had passed that part of the assessment without that information. If the additional marks possible from that part of the assessment for that student could not have elevated their outcome, then there is no point in offering a repeat assessment.

## Have obsessively guarded data handling

It is essential to ensure that the integrity of all results is rigorously protected. Staff should be clear of their responsibilities for safeguarding the security, confidentiality, accuracy and appropriate disclosure of results information. It is important to maintain high standards of attention to detail, a blame-free culture to encourage flagging of errors, and in particular, to ensure that accuracy is not compromised in order to return results quickly. There needs to be certainty of the accuracy of the data presented at any assessment meeting. Module leads (or equivalent) must be trained and have full understanding of all assessments and data checking processes, and be able to reassure members at the meeting. They should confirm that the processes to check the accuracy of data are appropriately rigorous, have several checkpoints to detect and correct any errors (e.g. in transposition), and have been followed.

## Defend the standards, not the students

Part A of the UK Quality Code requires university regulations to stipulate the threshold academic standard requirements and the basis for differentiating between grades ( QAA, 2013a). Thus, it is necessary to maintain consistent comparable threshold standards, but possible to set the standard higher than the minimum required by a professional body, or higher than other institutions. However, once a threshold standard has been decided it needs to be consistent and adhered to. For example a cut score, which has been based on the best available evidence, should not be eroded. Those who have failed to achieve a cut score, by however narrow a margin, have still failed to achieve the standard. Our duty is to ensure fairness to those who did comply with the standard required. Don’t make decisions giving the benefit of the doubt to a student, as this may disadvantage other students (e.g. in their ranking), and also compromise our duty to protect patient safety. Similarly, beware of making decisions which are considered to be in the student’s best interests (e.g. to not spend more time and money), as students could argue that they are best placed to judge this. Overall, it is best not to rely on a single predictor when making high stakes progression and award decisions ( Spurlock, 2006). Where some data is missing it may then be necessary to judge whether there is sufficient information to determine that the required learning outcomes have been achieved. It is also possible to pass and receive the credits, but not be permitted to progress or receive the award if there are outstanding concerns about fitness to practice.

## Have a robust process for consideration of individual student circumstances

It should be the student’s responsibility to declare, and be evaluated for, any disability that could impact their performance. Keep in mind the five key questions that the UK’s Office of the Independent Adjudicator asks itself in its duty to consider the Equality Act (2010): (1) is the student disabled?; (2) what provisions are being applied to the student?; (3) do those provisions place the student at a substantial disadvantage?; (4) what could be done to prevent that disadvantage?; and, (5) would it be reasonable to take those steps?

Extenuating (mitigating) circumstances (ECs) affect many students at one time or another. Students need to be aware to raise an EC request within a set time if they believe themselves to have been disadvantaged in any aspect of an assessment. To be taken into account when determining assessment outcomes an EC needs to be unexpected, outside the student’s control, and have the potential to negatively impact performance. There must be clear deadlines for EC submissions, and for retrospectively altering outcomes. Performance data associated with validated ECs, or any known pending investigation, should be considered atypical and thus excluded from any standard setting or ranking calculation that could have an impact on other students. Regardless of any EC, the results for an individual student must not be modified, as moderation of results can only occur for groups and with approval of the external examiner. If there is insufficient information to judge whether a student has achieved the learning outcomes then the student must be required to undertake additional assessment. Therefore, ECs do NOT absolve a student from undertaking an assessment. Thus, when required, ECs allow a student the opportunity to undertake an assessment when the circumstances are confirmed and resolved. Those with valid ECs who require additional assessment should receive an equivalent assessment to that missed. In this context, it is useful to distinguish an assessment ‘sitting’ (e.g. as either first sit/resit) from ‘attempt’ (i.e. as first/second etc.), and not prefix either with ‘final’. Where a reattempt at an assessment has been permitted because an EC has come to light late (for valid reasons) then the original attempt should be deemed null and void, and the reattempt should be counted as a first sitting (i.e. the attempt affected by the EC is not counted towards the finite number of sittings available). Where an EC is claimed to affect one assessment, be careful to review whether the EC should also be applied to other assessments occurring within the same time period. Where a student claims that they were not aware of a publicised assessment process (e.g. for submission of an EC within the required timeframe) this may be considered to indicate a lack of insight and raise concerns about fitness to attend clinical placements and engage with patients.

## Avoid conditional progression

The purposes of a progression decision are twofold: (1) to determine whether the student has demonstrated the required outcomes to be awarded credit for that stage; and (2), to determine whether there is sufficient evidence on the student’s performance to indicate that they are reasonably capable of successfully completing the next stage. Thus, it is necessary to judge using the evidence presented whether on the balance of probabilities that the student would successfully complete the next stage; or to similarly judge whether the student would successfully complete a repeat of the same stage. If not, then the student should be withdrawn from the programme. Conditional progression is possible, without being awarded credit or permitted further progression until they have satisfactorily completed the outstanding assessment. Thus a student could progress but with a warning letter of formal notice about additional requirements. The trailing of a small number of credits is relatively commonplace on standard degree programmes, where this is explicitly allowed by the regulations. However, it is advisable to finish the year they are in before being allowed to progress on clinical programmes because of patient safety concerns. Conditional progression can also create difficulties, where the student has not demonstrated readiness for material in next stage; and, will need to divert time from covering material in next stage in order to complete additional assessments, thus making that next stage harder (and vulnerable to appeal on grounds of inequity). Furthermore, if the student fails to meet the conditions of progression then it may be too late to permit a fair attempt at retaking the previous stage until the following year, which may necessitate interrupting studies for the remainder of the year. Thus if conditional progression is permitted it is essential to be clear what will happen if the student doesn’t fulfil the additional requirements.

## Have clear rules when further testing is permitted

It is necessary to determine how many times the same thing needs to be assessed to be confident a student is capable, and how many times an assessment can be repeatedly failed before being confident that a student is not capable. The assessment system must not permit students who fail one or a few assessments to then redeem at resit if that means that it has been easier to pass overall by spreading the assessment load. That would effectively lower the standard, and can be offset by having a rule that if one assessment is failed then all assessments must be retaken. To reduce the risk of double jeopardy, repeat attempts should be overseen by different assessors. Care should be taken to ensure that the standard of any additional assessment opportunity is equivalent to others. This may necessitate the opportunity not being made available until the next routinely scheduled iteration of that assessment, which may not occur until the next annual cycle. There should also be caps on resits and repeating years (to ensure every student can cope with contemporaneous workload intensity), combined with a maximum limit for the overall total duration of the programme (to ensure currency of content covered). Similarly, it must be made clear whether a student who interrupts their studies part way through a year is to return to assessments as first or second sit opportunities (and this decision might be based on the proportion of assessments they have previously attempted in that stage). If a student fails at a first attempt, then any subsequent attempts are to provide another opportunity to fulfil the requirements to pass for progression purposes and not to give an opportunity for a student to increase their ranking. Thus, any z-score calculations and ranking position should be based on first attempt scores. This is to ensure that those who pass first time are not unfairly disadvantaged by a student who fails first time then being able to achieve a higher rank by retaking an assessment. If a student is entitled to another attempt, but overall it would be impossible for them to achieve progression or award, then they should be offered an attempt as an opportunity for credits or remediation, but an attempt should not be offered if nothing can be gained to improve the outcome.

## Don’t set policy at progression or award meetings

Chapters A and B6 of the UK Quality Code are clear that progression requirements must be explicit, transparent and accessible in advance ( QAA, 2013a; QAA 2013b). Thus, curricular and regulatory changes are not to be determined at meetings intended to decide assessment outcomes. Decisions about assessment outcomes must be made in accordance with existing policies, although points of procedure can be referred for review in the future. Nevertheless, it may be appropriate to use discretion in cases where it is possible to demonstrate the learning outcomes have been achieved but where the regulations are unclear. When precedents are set, ensure that the reasons are documented clearly for future reference and any principle requiring curricular or regulatory review is referred to other committees as appropriate.

## Encourage challenge

Ensure that challenge is facilitated and that all the options are considered before reaching a decision. This helps protect against a student appealing that something was not taken into account, when it could have been. Make sure the feedback loop is closed so that the decision making process learns from appeals and OIA ‘case law’ ( OIA, 2017). It can also be useful to check that there are no assessment elements with unexpectedly high or low failure rates, or abnormal distributions in outcomes, which may indicate some assessments are more difficult than intended or have less support opportunities than others. Ideally, students who are unlikely to successfully complete a programme should be identified early. Thus, failure and exclusion rates should be highest in the earlier stages of a programme.

## Take Home Messages

There must be confidence that decisions follow due process, are fair, and achieve a comparable minimum threshold standard to other equivalent programmes. A student may fail due to: (1) a lack of aptitude (e.g. they may be underdeveloped, or have an incompatible core value such as caring about themselves more than others); (2) a lack of application (i.e. insufficient effort or inefficient use of effort; e.g. poor time management and procrastination); or (3) an adverse event (i.e. an extenuating circumstance which cannot be resolved within the timeframe of the programme; e.g. personal or social issues which are affecting their ability to perform). However no student should fail due to procedural irregularity, bias, or by any means which would not be comprehensible to a reasonable person.

## Notes On Contributors

STEVEN ASHLEY BURR, PhD, is an Associate Professor in Physiology and Deputy Director of Assessment for Medicine at Plymouth University Peninsula Schools of Medicine and Dentistry.

VEHID MAX SALIH, PhD, is an Associate Professor (Reader) in Oral & Dental Health Research and Deputy Director of Assessment for Dentistry at Plymouth University Peninsula Schools of Medicine and Dentistry.

THOMAS GALE, BMBS MEd, is a Clinical Associate Professor in Clinical Skills and Director of Assessment at Plymouth University Peninsula Schools of Medicine and Dentistry.

DAVID MOLES, PhD, BDS is a Professor of Oral Health Services Research and Director of Postgraduate Education and Research at Plymouth University Peninsula Schools of Medicine and Dentistry.

TERRY VALLANCE, is Head of Administration at Plymouth University Peninsula Schools of Medicine and Dentistry.

DAVID BRISTOW, PhD, is a Professor in Medical Education and Head of Peninsula School of Medicine at Plymouth University Peninsula Schools of Medicine and Dentistry.
