# Supplementary material for: Making progression and award decisions
Source: MedEdPublish (2016). 2017 Nov 7;6:200. [Version 1] doi: 10.15694/mep.2017.000200 (PMC10885224; doi:10.15694/mep.2017.000200)
Supplement: Peer review history

# Reviewer report: Reviewer response for version 1

**DOI:** 10.21956/mep.19386.r28412

**Published:** 2017-11-17

**Authors:** Julie Hunt

**Affiliations:** 1 Lincoln Memorial University

This review has been migrated. The reviewer awarded 5 stars out of 5 This manuscript provides a description of ideal policies and procedures surrounding student progress decisions. Although the authors primarily cite UK sources and policies, the essence of the article applies regardless of a college's location. As educators we remain challenged to assess and improve our assessments.

Reviewer Expertise:

NA

No decision status is available

# Reviewer report: Reviewer response for version 1

**DOI:** 10.21956/mep.19386.r28410

**Published:** 2017-11-07

**Authors:** Richard Hays

**Affiliations:** 1 James Cook University

This review has been migrated. The reviewer awarded 5 stars out of 5 This is a really helpful paper for those involved in managing assessment and progress decisions. Most appeals against decisions are lost on process errors, and the authors provide excellent guidance on getting that right. I have a couple of suggestions that might improve the paper a little. The first is that it is rather UK-centric, and yet the principles are universal. Some wording about adopting /conforming to the local institutional rules may help readers translate this into other contexts. The second is that not much is said about making assessment decisions more precise. While assessment practices are another whole topic, referring to getting that right (for example, using Standard Error of Measurement where possible for quantitative scores) may help readers understand that decisions are more fair and more defensible if assessment is done well.

Reviewer Expertise:

NA

No decision status is available

# Reviewer report: Reviewer response for version 1

**DOI:** 10.21956/mep.19386.r28411

**Published:** 2017-11-07

**Authors:** Trevor Gibbs

**Affiliations:** 1 AMEE

This review has been migrated. The reviewer awarded 5 stars out of 5 It was the educationalist David Boud who said that students can escape from bad teaching, but they cannot escape from bad assessment. Quality control of assessment is essential and this paper, although quite long, is an excellent example of how critical we need to be with our assessment procedures.I would happily recommend this paper to all those involved in assessment procedures as an essential read and although many of the references were UK -centric they were also an extremely useful set of resources.I specifically liked the last point regarding encourage challenge- something we probably don't do enough of in completing the assessment cycle and in moving ourselves forward in a very challenging area

Reviewer Expertise:

NA

No decision status is available

# Reviewer report: Reviewer response for version 1

**DOI:** 10.21956/mep.19386.r28409

**Published:** 2017-11-07

**Authors:** P Ravi Shankar

**Affiliations:** 1 American International Medical University

This review has been migrated. The reviewer awarded 5 stars out of 5 Assessment, progression and promotion decisions are critically important but have the potential for bias. This paper from the United Kingdom provides useful guidelines to academic leaders. Medical students should be ‘fit to practice’ as has been stressed by the authors and various accrediting and licensing agencies. I agree with the authors about the importance of protecting student anonymity so that decisions are not biased though this may be difficult in medical schools with small number of students. With practical examples the authors clearly describe the requirement that the assessment process should be fair to all students. Ensuring the integrity and reliability of assessment data could be a challenge in many situations. It is important that students are provided with an opportunity to challenge decisions. Though mainly focused on the United Kingdom this manuscript would be of interest to medical educators and academic leaders elsewhere.

Reviewer Expertise:

NA

No decision status is available
